# Investigation of Hemodialysis Patients’ Views on Thirst: A Mixed-Methods Study

**DOI:** 10.3390/healthcare14010056

**Published:** 2025-12-25

**Authors:** Ramazan Deniz, Bahar Çiftçi

**Affiliations:** 1 Department of Fundamental of Nursing, Institute of Health Sciences, Atatürk University, 25240 Erzurum, Türkiye; temmuzdeniz73@gmail.com; 2 Department of Fundamental of Nursing, Atatürk University Faculty of Nursing, Atatürk University, 25240 Erzurum, Türkiye

**Keywords:** hemodialysis, thirst discomfort, thirst management, mixed methods, nursing care

## Abstract

**Background/Objectives**: Thirst is a common and distressing symptom experienced by individuals undergoing hemodialysis. It can affect patients’ comfort, adherence to fluid restrictions, and overall quality of life. Understanding how patients perceive and cope with thirst is essential for developing evidence-based nursing interventions that enhance patient comfort and support adherence to care recommendations. **Aim:** To investigate the perceptions, experiences, and perceived factors related to thirst among hemodialysis patients using a mixed-methods design. **Methods:** This study adopted a convergent parallel mixed-method design. Quantitative data were obtained from 72 hemodialysis patients using the Thirst Discomfort Scale and a structured questionnaire. Qualitative data were collected through in-depth semi-structured interviews with 22 patients. Data were analysed using SPSS 23.0 for the quantitative phase and descriptive phenomenological analysis (Colaizzi’s method) for the qualitative phase to explore underlying perceptions and experiences. **Results:** Quantitative findings indicated substantial thirst discomfort, reflected by elevated Thirst Discomfort Scale and VAS scores. Qualitative findings highlighted persistent dry mouth, emotional distress, perceived loss of control, and coping strategies such as limiting fluids, distraction, oral rinsing, consuming cold items, and faith-based coping. On integration, qualitative narratives aligned with the high burden captured by quantitative scores, underscoring the multidimensional nature of thirst in hemodialysis. **Conclusions:** This study demonstrates that thirst in hemodialysis patients is a multidimensional experience encompassing physiological, psychological, and behavioural components. The findings highlight the need for individualised, holistic nursing approaches that extend beyond fluid restriction alone. **Relevance to clinical practice:** Nurses should adopt holistic approaches addressing emotional and spiritual dimensions of thirst management, providing individualised education and psychosocial support.

## 1. Introduction

Chronic diseases create a significant burden on health systems worldwide and negatively affect the quality of life of individuals. Nursing plays a critical role in the management of chronic diseases; therefore, the support provided by nurses in their relationships with patients directly affects the quality of care [1]. Nurses encourage patients to play a more active role in their care processes by providing education and resources tailored to their specific needs. In particular, improving self-care skills directly affects patients’ quality of life, and nurses are essential professionals in guiding this process [2].

Chronic renal failure (CRF) is a chronic disease with high morbidity and mortality rates affecting health services and patient care [3]. It has been reported that its prevalence ranges from 8% to 16% worldwide [4]. CRF management requires multidisciplinary care [5]. In chronic renal failure, the hemodialysis treatment method is widely used before kidney transplantation [6]. The primary task of nursing care in hemodialysis is continuous monitoring of patients before, during, and after the dialysis session to detect complications such as intradialytic hypotension, muscle cramps, and other haemodynamic changes [7]. However, hypervolemia is one of the most critical complications encountered during hemodialysis. Various prevention strategies, such as fluid management and oedema control applied by nurses to manage such conditions, improve patient care and ensure fluid balance [8].

One of the priority issues in the care of hemodialysis patients is maintaining an appropriate fluid balance. Patients with end-stage renal failure experience difficulties with fluid control due to impaired renal function, making fluid volume management a common challenge in clinical practice [9]. The concept of “dry weight” is central to fluid management; it is the patient’s target weight after excess fluid has been removed during dialysis. Accurate estimation of dry weight is crucial because it enables nurses to set appropriate ultrafiltration targets during treatment sessions. Failure to maintain appropriate fluid levels can lead to complications such as hypertension, pulmonary oedema, and cardiovascular problems, all of which are risks associated with excessive extracellular fluid volume [10]. Patient education regarding fluid restriction is crucial for preventing interdialytic weight gain (IDWGA). Nurses are responsible for educating patients about fluid intake limitations and the importance of adhering to protocols to avoid volume overload. As part of fluid management strategies, nurses should guide patients in recognising sources of hydration, including foods with high water content, such as fruits and soups, and emphasise that these contribute to overall fluid intake [11]. Thirst management in hemodialysis patients involves processes that include the prevention of complications such as excessive fluid retention, interdialytic weight gain, and directly affect patient compliance with treatment [12].

Thirst is a prevalent symptom in hemodialysis patients, with a prevalence ranging from 67% to 97% [13]. Following an increase in dietary salt intake, extracellular fluid osmolality rises, thereby increasing water intake. However, because renal function is compromised and urine production is absent or very low, fluid or food intake leads to fluid overload and hyponatremia. As a result, thirst occurs at the end of the haemodialytic session and in the following hours [14]. Previous studies have described several physiological and psychological contributors to thirst, including low potassium, acute increases in plasma urea, hyperglycaemia, plasma sodium concentration, angiotensin II, and psychological factors [15]. Dealing with the symptom of thirst requires person-centred strategies. In developing strategies to manage thirst in settings such as hemodialysis units, the counselling and educational roles of nurses working in these units become evident. Since nurses are generally the closest professional group to patients, they should take an active role in informing patients, following evidence-based practices on appropriate interventions and making joint decisions with the patient [16]. Although previous studies have extensively examined thirst in hemodialysis patients, most have focused either on quantitative symptom measurement or on qualitative experiences alone. There remains a limited understanding of how objectively measured thirst-related discomfort aligns with patients’ lived experiences and coping strategies within an integrated mixed-methods framework.

This study aims to examine patients’ thirst levels and perceptions during hemodialysis by integrating quantitative assessments of thirst-related discomfort with qualitative exploration of patients’ experiences.

**Research Questions:**

What are the thirst-related discomfort levels of patients receiving hemodialysis treatment?

What situations and conditions do patients perceive as increasing their thirst discomfort?

What coping strategies are used to manage thirst in patients undergoing hemodialysis?

## 2. Materials and Methods

### 2.1. Research Type

In this study, a convergent parallel mixed-methods design was employed, in which quantitative and qualitative data were collected simultaneously, analysed separately, and the results were combined to develop a holistic understanding [17]. During the planning and reporting of the research, the “Good Reporting of A Mixed Methods Study (GRAMMS)” checklist, which aims to improve the quality of mixed-methods studies, was used as a guide [18].

The integration of quantitative and qualitative findings occurred during the interpretation and discussion stages, employing a narrative-weaving approach. Quantitative descriptive results (e.g., Thirst Discomfort Scale and VAS scores) were compared alongside qualitative themes derived from Colaizzi’s analysis to generate meta-inferences regarding the multidimensional nature of thirst in hemodialysis patients. Integration was further supported by comparing qualitative codes with TDS sub-dimensions to identify convergence and divergence.

### 2.2. The Research’s Population and Sample

The study was conducted in the Hemodialysis Unit of Ağrı Training and Research Hospital. Data were collected from patients receiving hemodialysis treatment between February and November 2025. The study population consisted of 80 patients. A priori power analysis in G*Power 3.1.9.4 (Heinrich Heine University Düsseldorf, Düsseldorf, Germany) was used to determine the sample size. In line with this result, the minimum number of patients required for the study was 65, based on an effect size of 0.67, a 95% confidence interval, a 0.05 significance level, and a power of 0.95 [19,20]. For this study, a sample size of 72 patients was calculated, considering a potential 10% data loss. After excluding three patients using diuretics/antipsychotics and five pilot participants, the final quantitative sample comprised 72 patients. According to the sample size for the qualitative data of the research, the number of participants required to contribute to the research is stated as 20–40 [21]. Qualitative interviews were conducted with participants who met the sampling criteria for the qualitative research component of the study, alongside the quantitative participants. Given sufficient data saturation, the qualitative component of the study was completed with 22 patients. Qualitative participants were purposively selected from the quantitative sample to ensure variation in thirst severity and treatment duration.

### 2.3. Inclusion and Exclusion Criteria

Inclusion Criteria

- Aged 18 years and older;
- Being diagnosed with Chronic Renal Failure;
- Not having any psychiatric illness;
- Being open to communication;

Exclusion Criteria

- Experiencing deterioration in general condition;
- Taking diuretics, antipsychotics, or opioids.

Patients using diuretics, antipsychotics, or opioids were excluded because these medications may alter fluid balance, salivary secretion, thirst perception, and cognitive processes, potentially confounding both quantitative thirst measurements and qualitative experiential reports.

### 2.4. Data Collection Tools

#### 2.4.1. Data Collection Tools to Be Used in the Research

In the study, the Introductory Information Form, including questions on sociodemographic characteristics; the Thirst Discomfort Scale; the Visual Analogue Scale; and the Semi-structured Interview Form, prepared for in-depth interviews, were used. All data collection tools were administered in Turkish. The Thirst Discomfort Scale was initially developed and validated in Turkish, and the Introductory Information Form and the Semi-structured Interview Form were also created in Turkish by the researchers. Therefore, no translation, back-translation, or cultural adaptation procedures were required in this study.

##### Introductory Information Form

This form consisted of 18 questions regarding age, BMI, gender, marital status, educational status, place of residence, employment status, income status, smoking, alcohol, and coffee consumption, daily fluid intake, presence of chronic diseases other than CRF, duration of hemodialysis treatment, itching status, and mobility status.

##### Thirst Discomfort Scale

The Thirst Discomfort Scale comprises 12 items and three sub-dimensions, developed by Çiftçi et al. [22]. The Thirst Discomfort Scale is calculated over the total item score. The scores that can be obtained in the “Intraoral Movements” sub-dimension vary between 5 and 25. The scores that can be obtained in the “Psychological Movements” sub-dimension range from 4 to 20. In contrast, the scores that can be obtained in the “Extraoral Movements” sub-dimension vary between 3 and 15. The minimum score on the scale is 12, and the maximum is 60. As scores on the sub-dimensions increase, the level of related discomfort also increases. As the total score obtained from the scale increases, the level of discomfort associated with thirst in patients also increases. The overall Cronbach’s Alpha value of the scale was determined to be 0.886 [22]. In this study, the Cronbach’s alpha for the Thirst Discomfort Scale was 0.917.

##### Visual Analogue Scale (Thirst Level)

VAS was used to quantify subjective data on participants’ thirst levels and to corroborate the findings expressed verbally. This scale comprises the following response options: “0: I do not experience thirst”, “5: I experience moderate thirst”, and “10: I experience high thirst”.

##### Semi-Structured Interview Form

The qualitative data for the study were collected using a semi-structured interview form developed in parallel with the quantitative scale. As a data collection tool, the draft semi-structured interview form, developed by the researcher based on the literature, consisted of eight questions. The first draft of the semi-structured interview form was sent to 10 academicians, experts in their respective fields and working in nursing, for semantic and content evaluation. This form, organised in line with expert opinions, consists of 7 questions in total, including six main questions and one probe question.

### 2.5. Data Collection Procedure

Before data collection, participants were informed in detail about the study’s purpose and methods, and then they provided their verbal and written consent. A convergent parallel design was employed in the mixed-methods approach, collecting both quantitative and qualitative data simultaneously. Quantitative data were collected through face-to-face interviews with patients undergoing hemodialysis treatment, using the Introductory Information Form, Thirst Discomfort Scale, and Visual Analogue Scale (VAS). The researcher read the questions, and the participants’ answers were marked; any incomprehensible points were explained. The forms were then completed in full. Quantitative data collection took approximately 5 to 10 min. Face-to-face, in-depth interviews conducted by the researcher lasted 15–30 min and were recorded on a voice recorder. Each participant was assigned a code name (K1, K2, …, K22) and then transferred to the computer environment. Although quantitative data collection started slightly earlier, qualitative interviews were conducted during the same data collection period, and both datasets were treated as equally weighted and independently analysed prior to integration.

### 2.6. Characteristics of the Place of Interview

To collect quantitative and qualitative data for the study, an environment was created in which patients could speak quietly and comfortably, after ensuring their safety and comfort, and measures were implemented to supervise patients at risk of falling by a nurse or student nurse. The sites selected for data collection were quiet environments with adequate heating, lighting, and ventilation.

### 2.7. Pilot Application

A pilot study was conducted with five patients to assess clarity and feasibility. No major revisions were required. Pilot participants were excluded from the final analysis.

### 2.8. Data Evaluation

#### 2.8.1. Evaluation of Quantitative Data

Data analysis was performed using SPSS 23 (IBM Corp., Armonk, NY, USA). Frequency and percentage calculations, as well as mean and standard deviation values, were used to evaluate the data related to descriptive characteristics [23]. Inferential statistical analyses were not performed, as the quantitative component was designed to provide descriptive context for the qualitative findings rather than to test associations. Although inferential analyses were not the primary aim, the quantitative results provided a descriptive profile that informed purposeful sampling for the qualitative phase and strengthened integration.

#### 2.8.2. Evaluation of Qualitative Data

The qualitative data were systematically analysed using Colaizzi’s descriptive phenomenological analysis method [24]. This approach aims to understand the essence of participants’ thirst experiences in depth and to generate clinically meaningful themes. The analysis process was carried out in the following seven stages: (1) transcription and detailed examination of the interview recordings, (2) systematic identification of meaningful statements, (3) transformation of these statements into conceptual meanings, (4) formation of themes by grouping similar content, (5) development of a comprehensive definition based on the themes, (6) revealing the basic structure of the thirst phenomenon, and (7) verification with feedback from participants. As a result, two main themes were identified: the effects of Hemodialysis on Thirst and Problems Experienced, and Time, Factors, and Coping Experiences with Thirst. Colaizzi’s method enhanced the scientific rigour of the study by enabling a multidimensional understanding of thirst grounded in patients’ lived experiences, complementing the quantitative findings. To minimise potential researcher bias, several strategies were employed. All interviews were audio-recorded and transcribed verbatim to ensure accuracy. Data analysis followed Colaizzi’s structured analytic steps, which provided a transparent and systematic framework. Reflexive notes were kept throughout the analysis process to enhance analytical awareness. In addition, the research team reviewed and discussed preliminary themes, and participant validation (member checking) was used to confirm the credibility of the findings.

### 2.9. Ethical Principles of the Research

Ethics committee permission was obtained from Atatürk University Non-Interventional Clinical Research Ethics Committee with the number B.30.2.ATA.0.01.00/464 for the conduct of this research. To conduct the study at the Hemodialysis Unit of Ağrı Training and Research Hospital, the necessary institutional permission was obtained from the Ağrı Provincial Health Directorate under number 24.09.2024/164. Additionally, written and verbal consent were obtained after each participant was informed of the study’s methods and rationale. Those who wished to participate in the study were included. To protect individual rights in research, full compliance with the Declaration of Helsinki on Human Rights was ensured. The nurse in charge, the physician in charge of the unit, and other health personnel working in the hemodialysis unit were informed about the research.

## 3. Results

The sociodemographic and clinical characteristics of the patients receiving hemodialysis are presented in Table 1. The sample included predominantly middle-aged and older adults, with common comorbid conditions such as hypertension and notable levels of thirst severity.

The distribution of the mean scores of the Thirst Discomfort Scale and its sub-dimensions is presented in Table 2. Overall, patients demonstrated high levels of thirst-related discomfort across intraoral, psychological, and extraoral dimensions.

In the qualitative component of the study, 22 patients receiving hemodialysis participated. Participants ranged in age from 20 to 71 years and included both male and female patients with varying clinical characteristics. The duration of hemodialysis treatment ranged from 2 months to 15 years, and daily fluid intake ranged from 1 to 10 glasses. Most participants reported high thirst severity, with the majority scoring 9 on the Visual Analogue Scale, indicating pronounced thirst.

Qualitative analysis revealed two main themes related to patients’ thirst experiences during hemodialysis, encompassing six sub-themes and 27 codes (Table 3).

Theme 1. Effects of Hemodialysis on Thirst and Problems Experienced

Three sub-themes, namely “Psychological Effects”, “Pre-dialysis Problems”, and “Post-dialysis Problems” were identified regarding the effects of hemodialysis on thirst and the problems experienced (Table 3).

Sub-theme 1. Psychological Effects

In the sub-theme of psychological effects, three codes were determined as “fatigue”, “fear”, and “inability to socialise” (Table 3). Patients stated that they felt psychologically tired due to thirst before and after hemodialysis, they were afraid of drinking water, they were fearful of bloating because they could not excrete when they drank water, they were worried about fainting during hemodialysis due to being overweight and low blood pressure. In addition, the patients reported avoiding attending friends’ invitations or meeting with their families, and that they could not socialise because they could not tolerate the food at such events and believed their need for water would increase after eating.

P3, “I can’t take my child to the park by myself because my blood pressure may drop, I may faint… I haven’t been able to go to my father’s house for a long time… I never urinate. I never urinate; it is a bit difficult for me, I feel bored, I get bored.”

P6, “If we drink less fluid, we are not very uncomfortable during dialysis, but when we drink a lot of water or tea, we sometimes feel uncomfortable during dialysis. Blood pressure drops, and we feel weak and tired. When I go out with friends, I can’t help but drink a lot of tea, which is bad for me.”

P20, “I am usually tired and weak because I cannot drink water.... Now I have reduced drinking water out of fear. When I do not pay attention, dialysis is like torture for me.”

Sub-theme 2. Pre-dialysis Problems

In the sub-theme of pre-dialysis problems, five codes were determined as “dry mouth/throat”, “inability to drink water”, “excessive thirst”, “bloating/ difficulty in movement”, and “inability to excrete” (Table 3). The majority of patients reported dryness of the mouth and throat due to pre-dialysis bloating, discomfort from fluid accumulation, and reluctance to drink water because they were unable to excrete it. They stated that they were extremely thirsty because they had not taken enough fluid daily, and they needed to fulfil their nutritional needs. They said that when they took too much fluid before dialysis, they had bloating because they could not excrete, and therefore, they had difficulty moving.

P1, “I swear I drink a lot of water. After I drink, I can’t go to the toilet, and because I can’t, I swell up. After that, when I swell up, I have a lot of difficulty; I can’t move.”

P8, “When I am thirsty, my mouth and throat get parched. I am very thirsty, but I cannot drink now, I swell up immediately.”

P11, “I am very uncomfortable because I come to dialysis with excess weight. I urinate very late; I have a lot of difficulty, but I cannot stop drinking water.”

P18, “I want to drink water, but I don’t dare because it comes from my nose in dialysis... My mouth and throat get dry immediately. If I drink a little, it does not stop. When I drink too much, my body swells.”

Sub-theme 3. Problems after dialysis

In the sub-theme of post-dialysis problems, four codes were determined as “dry mouth”, “excessive thirst”, “dizziness/weakness”, and “drop in blood pressure” (Table 3). The majority of patients reported experiencing dry mouth after dialysis. In addition, they stated that their desire to drink water increased excessively after dialysis, and they encountered problems such as dizziness, weakness, and low blood pressure.

P5, “Firstly, my mouth gets dry, I want to drink some water and rest. My blood pressure drops after dialysis. I want to rest, but I can’t.”

P7, “When I come out of dialysis, I feel dizzy, my mouth gets dry, and I drink more water at that time.”

P13, “My throat and mouth get dry when I feel thirsty. Other than that, if I see someone drinking water, I immediately want to drink it too.”

P22, “My insides are burning. Then I want to drink a lot of water. My mouth is always dry anyway.”

Theme 2. Time, Factors, and Coping Experiences Related to Thirst

Three sub-themes, namely “Period of Thirst”, “Factors Increasing Thirst”, and “Actions Taken to Quench Thirst” were identified for time, factors, and coping experiences related to thirst (Table 3).

Sub-theme 4. Period of Thirst

In the sub-theme of the period when thirst was experienced, six codes were determined as “after the meal”, “night”, “evening”, “morning”, “noon”, and “summer months” (Table 3). The majority of the patients stated that their desire to drink water increased excessively after lunch and dinner. They also said that they woke up from sleep to drink water, especially at night, and that they were very thirsty in the evening because of the dinner they had eaten. Apart from this, they stated that they were very thirsty at noon, especially in the summer.

P4, “I mean, sometimes when I wake up in the middle of the night, I feel very thirsty. At those times, I pay attention, I try not to drink too much, but I also get very thirsty in the evenings.”

P10, “I mean, I usually get very thirsty after dinner. Apart from that, sometimes when I wake up at night, I want to drink water, but I don’t. I get very thirsty, especially when I work in summer.”

P14, “I get thirsty mostly in the evenings. After meals, especially if they are salty, I become thirsty immediately. At night, I sometimes wake up because I’m thirsty. My mouth is dry.”

P21, “I feel thirstier more between noon and evening. I also crave water when I wake up in the morning.”

Sub-theme 5. Factors Increasing Thirst

In the sub-theme of factors that increase thirst, four codes were identified: “moving”, “fatigue”, “salt intake”, and “meat consumption” (Table 3). The patients reported that their thirst increased and they tended to drink more water when they were more active, as well as on days when they felt tired. In addition, they reported feeling thirstier after consuming excessive salt in their meals during the day and after consuming meat.

P9, “My thirst increases when I move. It is the same when I do housework. Other than that, I get thirsty when I eat a meaty meal.”

P12, “When I get tired, for example, if I don’t drink water, I don’t feel relieved. I get very thirsty, especially when I eat meaty food. I get very thirsty, especially when the dinner is a bit salty.”

P17, “But I get thirsty if I move, of course.... Recently, we purchased rock salt and added a small amount to our food. This makes me thirsty.”

Sub-theme 6. Things done to quench thirst

In the sub-theme of what to do to quench thirst, five codes were determined as “drinking cold water”, “eating fruit”, “drinking tea”, “rinsing the mouth with water”, and “drinking buttermilk” (Table 3). Patients reported that when thirsty, they mostly preferred cold water and that they suppressed their desire to drink by eating fruit. In addition, the patients reported that they quelled their thirst by drinking tea, rinsing their mouths with water, and drinking ayran. Some interview examples for the codes determined in sub-theme 6 are as follows:

P2, “I mean, I want to drink cold water all the time, because I feel a burning and flare-up inside. Eating fruit is especially good for quenching my thirst. I put them in the fridge, and I can eat them after they cool down.”

P11, “I drink cold things. Water, fruit, and buttermilk are good for me.”

P15, “Drinking tea at work breaks thirst. Additionally, I sometimes rinse my mouth with water. I can’t drink warm water, it has to be ice cold…”

P16, “But I feel relieved when I rinse my mouth with water. Also, cold water is delightful for dialysis patients and me too.”

P19, “When I drink tea, I stop feeling a little tired. And the water will be ice cold so that I can drink it.”

When quantitative and qualitative findings were integrated, high Thirst Discomfort Scale and VAS scores were reflected in patients’ narratives describing persistent dry mouth, emotional distress, fear of fluid intake, and behavioural coping strategies. In addition, the variability observed in the quantitative sub-dimension scores, particularly within the Intraoral Movements domain, was reflected in the qualitative interviews. Patients who described more intense oral dryness, burning sensations, and persistent mouth discomfort reported frequent use of immediate sensory coping strategies such as drinking cold water, rinsing the mouth, consuming cold foods, or drinking tea. In contrast, participants who described relatively less intense intraoral discomfort tended to report behavioural regulation strategies, including consciously limiting fluid intake, delaying drinking, or distraction.

## 4. Discussion

The findings of this study, which examined hemodialysis patients’ views on thirst, were discussed in the context of the literature as follows.

Fluid restrictions in hemodialysis patients are implemented as a clinical necessity to prevent complications such as interdialytic weight gain and hypertension. These restrictions lead to a constant feeling of thirst and significantly impact the quality of daily life for patients [25]. The high level of scale and VAS results obtained in the study reveals the extent of the discomfort caused by the need to restrict fluid intake. In the literature, it has been reported that hemodialysis patients experience widespread and intense thirst [12,14,15]. In particular, the scale results indicate that thirst is not only a physiological symptom but also a complex one that affects quality of life. Qualitative data also support this, revealing that the need to suppress patients’ desire to drink water affects various aspects of their daily lives, including their meal patterns and social interactions. Bruzda-Zwiech et al. state that fluid restrictions, although a clinical necessity, create a significant feeling of thirst and are challenging to manage [12]. Bossola et al. emphasised that a substantial proportion of hemodialysis patients experience persistent thirst, which may adversely affect treatment adherence and quality of life [14]. Fernandes et al. revealed that high sodium intake triggers the thirst response by increasing plasma osmolarity, highlighting the critical role of dietary management [26]. Hong et al. stated that anxiety about fluid restrictions increases patients’ awareness of thirst, magnifying their psychological burden and may affect treatment compliance [27]. Pan et al. noted that thirst is a crucial symptom that necessitates special consideration in patient-centred care [25]. The high scale and VAS scores in the study reflect the difficulties patients encounter during treatment and the challenges of managing thirst. Qualitative findings indicate that thirst is a continuous aspect of the treatment process rather than a temporary discomfort, and is deeply intertwined with patients’ subjective experiences.

The high mean scores obtained in the Intraoral Movements sub-dimension of the Thirst Discomfort Scale in the study indicate that thirst discomfort is experienced intensely, particularly in the mouth and throat, among hemodialysis patients. In the qualitative component, interviewees were purposively selected to reflect variation in thirst severity, including patients with relatively higher and lower intraoral discomfort profiles as indicated by the Thirst Discomfort Scale. Participants describing more intense oral dryness and burning sensations consistently reported immediate, sensory-based coping strategies such as drinking cold water, rinsing the mouth, or consuming cold foods, whereas those reporting relatively milder oral discomfort more often emphasised behavioural regulation strategies, including consciously delaying fluid intake, limiting consumption, or using distraction. Although formal subgroup or inferential comparisons were not conducted, this pattern suggests that the observed variability in intraoral movement scores reflects meaningful differences in lived experience and coping behaviours rather than unexplained statistical outliers. This integrated interpretation strengthens the clinical meaning of IM scores as reflecting oral discomfort that shapes coping preferences. Although hemodialysis treatment removes excess fluid and waste by taking over the filtration function of the kidneys, it requires severe fluid restrictions [25]. These restrictions increase plasma osmolality, and physiological processes, such as sodium retention, stimulate the sensation of thirst and dryness of the oral mucosa [12,26]. Qualitative findings also support this situation, revealing that patients reported prominent symptoms, including dry mouth and throat, difficulty drinking water, and excessive thirst, particularly before dialysis. These experiences align with high scores on the scale subscale, indicating that the inability to drink water is not only a physical limitation but also a source of persistent discomfort in daily life. Similarly, in the literature, Bossola et al. emphasised that the feeling of thirst in hemodialysis patients is expressed through somatic symptoms, such as drying of the oral mucosa and decreased saliva production [14]. Bruzda-Zwiech et al. stated that sodium restriction is crucial for maintaining fluid balance and that excessive sodium intake increases plasma osmolality, thereby triggering thirst and a dry mouth [12]. Fernandes et al. noted that the difficulties of managing dietary sodium are among the primary factors that increase the sensation of thirst [26]. It has also been reported that sudden changes in electrolytes and fluids during dialysis can increase dryness in the mouth, exacerbating the desire to drink water and causing unpleasant oral symptoms in patients [28,29]. These findings reveal that thirst is not only a side effect of fluid-restriction protocols but also a symptom arising from complex physiological and daily-life dynamics associated with the treatment process.

The high scores obtained on the Extraoral Movements sub-dimension of the Thirst Discomfort Scale in the study indicate that the perception of thirst in hemodialysis patients is not merely a subjective feeling but also manifests as significant behavioural and reflexive responses. This sub-dimension assesses automatic responses to alleviate thirst, such as lip licking, tongue sticking, and mouth movements, and objectively measures physiological responses to dry oral mucosa. In the qualitative data, symptoms such as dry mouth, excessive thirst, dizziness, and weakness were prominent after dialysis; in particular, reports of dry mouth and thirst were consistent with this sub-dimension of the scale. Patients stated that they experienced intense thirst after dialysis due to sudden fluid withdrawal and an increase in plasma osmolality, and that they developed involuntary behavioural responses to reduce this feeling. In the literature, it is emphasised that the rapid reduction of intravascular volume during hemodialysis triggers intense thirst by stimulating osmoreceptors [12,25]. Fernandes et al. stated that sodium balance is an essential factor in determining the severity of thirst during dialysis [26]. Additionally, complications such as dizziness, weakness, and low blood pressure can occur due to rapid ultrafiltration and sudden changes in intravascular volume, which negatively affect treatment tolerance by increasing thirst perception [30]. These findings suggest that thirst management should not focus solely on fluid restrictions; strategies that address physiological responses and patient experiences holistically should be developed, including planning the ultrafiltration rate, sodium profile, and dialysis duration according to patient tolerance.

In the study, high scores in the Psychological Actions subscale of the Thirst Discomfort Scale indicate that thirst is not only a physiological symptom but also a significant psychological burden in hemodialysis patients. This subscale measures the internal tension and neurovegetative responses associated with thirst, including symptoms such as fatigue, restlessness, anxiety, and dizziness. A high scale score indicates that patients experience thirst as a significant stress response and emotional burden. Patients report that thirst has become a chronic source of stress in daily life, leading to multidimensional reactions such as fatigue, anxiety, and social withdrawal. Fatigue is one of the most frequently reported symptoms in hemodialysis patients and is exacerbated by the physiological demands of the dialysis process, anaemia, nutritional deficiencies, and metabolic stress [14,31]. Feelings of anxiety and fear are associated with difficulties in compliance with fluid restrictions and risks of complications. Fernandes et al. state that fluid and sodium restrictions are not only physiological but also an essential source of psychological stress [26]. Hong et al. stated that anxiety about fluid restrictions makes the perception of thirst more acute and may negatively affect treatment adherence [27]. Lack of socialisation is one of the direct effects of thirst and treatment requirements on social life. Patients tend to withdraw from social environments due to frequent dialysis sessions, fluid restrictions, and dietary regulations. Alexopoulou et al. noted that a lack of social support can reduce quality of life and exacerbate psychological problems by increasing feelings of loneliness [32]. Fernandes et al. emphasised that fluid restrictions may remove patients from social dining activities [26]. These findings, which are also supported in the literature [12,25], reveal that thirst is a multidimensional stressor in hemodialysis patients, and that management should be addressed not only in physiological but also in psychological and social aspects. It is essential to develop a patient-centred, multidisciplinary approach that accounts for patients’ emotional responses, mental health, and social experiences.

In the qualitative findings of the study, within the theme of Time, Factors, and Coping Experiences Related to Thirst, it was determined that patients experienced thirst more intensely, particularly after meals, at night, in the evening, in the morning, at noon, and during the summer. These periods are associated with both physiological and environmental factors. Increased thirst after meals is often associated with patients not adhering to sufficient salt restrictions, while decreased salivation at nighttime is thought to contribute to dry mouth and further exacerbate thirst. During the summer months, sweating increases fluid loss and heightens the sensation of thirst. High sodium intake between hemodialysis treatment sessions, dry mouth due to fluid restriction, and psychological stress factors also stand out as important factors that increase the feeling of thirst [25]. The sodium content of dialysate during treatment can directly affect thirst; lower sodium levels have been associated with reduced thirst and better fluid management.

Furthermore, the withdrawal of waste and fluids during dialysis can temporarily reduce the perception of thirst, while high ultrafiltration or rapid dialysis can trigger thirst [33]. However, it has been shown that thirst usually increases again 4–6 h after dialysis is completed [34]. Unlike the existing literature, this study is believed to contribute to the development of individualised fluid management strategies in clinical practice by highlighting the specific periods during which hemodialysis patients experience thirst.

In the qualitative findings of the study, within the theme of Time, Factors, and Coping Experiences Related to Thirst, the main factors reported by patients as increasing thirst were movement, fatigue, salt intake, and meat consumption. Thirst in hemodialysis patients is a complex symptom that results from the interaction of physiological, dietary, and psychological factors. Although restricting fluid intake is a necessary approach to prevent complications such as hypertension and pulmonary oedema, it can negatively affect quality of life by increasing thirst in patients [15]. High sodium intake is a significant factor in the exacerbation of thirst. Sodium stimulates the thirst response by increasing plasma osmolarity. Fernandes et al. emphasise that excessive sodium consumption elevates thirst levels, and dietary management plays a critical role in controlling thirst [26]. Psychological factors may also influence the perception of thirst. Hong et al. state that anxiety about fluid restrictions may increase awareness of thirst, thereby complicating patients’ emotional well-being and treatment adherence [27]. The removal of fluids by ultrafiltration during and after hemodialysis may also trigger thirst. Chen et al. demonstrate that this thirst can persist for several hours after dialysis [34]. Hormonal factors also play a role in the occurrence of thirst; high levels of angiotensin II, via the renin-angiotensin system, can increase the perception of thirst [35].

Furthermore, Debnath et al. state that physical inactivity is linked to increased fatigue, which may exacerbate the sensation of thirst [36]. Johansen et al. noted that patients with fatigue may struggle to comply with fluid restriction instructions [37]. These findings suggest that thirst is a multidimensional issue shaped by fluid restrictions, dietary practices, and patients’ subjective experiences fatigue, and physical activity in hemodialysis patients. Therefore, it is essential to address thirst management through a holistic model that encompasses education, physiological interventions, and supportive care.

In the qualitative findings of the study, the most common strategies under the themes of Time, Factors, and Coping Experiences Related to Thirst were drinking cold water, eating fruit, drinking tea, rinsing the mouth with water, and drinking buttermilk. This finding indicates that hemodialysis patients develop simple, accessible, and personally preferred strategies to combat thirst. Thirst-reducing practices not only provide behavioural relief but also target sensory and neurovegetative responses. For example, sucking on ice cubes or sugar-free candy can alleviate dry mouth by stimulating salivation and providing temporary relief from the perception of thirst [38,39]. Similarly, chewing sugar-free gum has been reported to reduce dry mouth by increasing saliva production [40]. However, meta-analyses of the efficacy of these methods, such as chewing gum, have shown inconsistent results and have not yielded a significant reduction in Dialysis Thirst Inventory scores. Dietary management is also a critical coping strategy. High sodium intake increases plasma osmolality, exacerbating thirst and leading to fluid retention. Therefore, it is emphasised that low-sodium diet programmes are effective in managing thirst [15,41]. It is recommended that patients avoid overly salty foods and prefer foods with high water content, such as fruits [42]. Non-pharmacological interventions are also found in the literature. For example, oral care with menthol solutions has been shown to alleviate dryness by improving the oral mucosa [43]. In addition, Keskin and Taşci found that acupressure can reduce the severity of VAS thirst by increasing saliva production. They reported that patients developed conscious strategies, such as consuming lemon drinks or cold drinks and sipping slowly [44]. Planning fluid intake is also a critical approach. Daily fluid tracking charts or record-keeping can help patients recognise their intrinsic thirst factors and become more conscious of fluid management [45]. However, intervention studies have shown that even distributing fluid intake over time can reduce thirst scores [46].

On the other hand, alternative sensory interventions such as ice cubes and frozen strawberries have also been evaluated in the literature. For example, Hudiyawati and Suswardany found that frozen strawberries reduced thirst intensity more than ice cubes [47]. Taken together, these findings suggest that thirst is not only a physiological symptom in hemodialysis patients, but also a multifaceted experience with dietary, behavioural, psychological, and sensory dimensions. Therefore, its management should not be limited to a single type of strategy. The most significant contribution of the study is that it underscores the need for patient-centred, individualised interventions in clinical care by providing detailed insights into patients’ coping strategies. However, the literature and this study’s findings also show limited and heterogeneous evidence regarding the effectiveness of the methods employed. For example, some techniques, such as chewing gum, may be practical to varying degrees among patients [48]. Therefore, it is of great importance to develop innovative and evidence-based non-pharmacological strategies using larger samples, multicenter studies, and methodologically sound approaches.

The findings of this study provide clinically relevant insights for nursing practice in hemodialysis units. In particular, the finding that patients reported tea drinking as a common coping strategy, with a mean daily tea/coffee consumption of 3.22 cups, has important clinical implications. According to international hemodialysis guidelines, all beverages—including tea and coffee—contribute to total daily fluid intake and should be considered in fluid restriction plans. Therefore, nurses should assess patients’ beverage preferences and provide individualised counselling, explicitly clarifying that tea and coffee contribute to the total daily fluid allowance and may increase the interdialytic fluid burden if not accounted for. This level of habitual tea and coffee consumption may therefore pose a risk for unintentional excess fluid intake, particularly when patients do not perceive these beverages as part of their fluid allowance.

In addition, the qualitative findings indicating that thirst intensifies after meals, during the evening, and at night suggest the importance of time-specific interventions. This aligns with clinical recommendations supporting the use of sensory-based, low-volume strategies—such as oral rinsing, ice chips, and cold foods—during peak thirst periods to reduce discomfort without increasing fluid burden. Integrating these strategies into routine nursing counselling may help patients manage thirst more effectively while maintaining adherence to liquid restrictions.

Furthermore, patients’ reports of salt intake as a key trigger of thirst reinforce existing guideline recommendations that emphasise dietary sodium reduction as a cornerstone of thirst and fluid management in hemodialysis patients. Nurses play a critical role in translating these recommendations into practical guidance, including label reading, meal planning, and culturally appropriate dietary advice.

Future research should prioritise multicentre, longitudinal designs with larger and more diverse samples to enhance the generalisability of findings on thirst in hemodialysis patients. In addition, intervention-based studies evaluating nursing-led, non-pharmacological strategies—such as structured oral care protocols, dietary counselling, behavioural thirst-management interventions, and sensory-based approaches—are warranted. Integrating quantitative outcomes with qualitative patient experiences may further support the development of personalised, evidence-based thirst management models in clinical practice.

### Limitations of the Study

Despite its contributions, this study has several limitations. First, as the study was conducted in a single centre, the findings may not be generalisable to broader hemodialysis populations. In addition, participants shared similar cultural and socioeconomic characteristics, which may limit the transferability of the results to different social contexts.

Furthermore, certain dialysis-related clinical variables that may influence thirst perception—such as dialysate sodium concentration, ultrafiltration volume, residual urine output, and interdialytic weight gain—were not collected or analysed in this study. Although the absence of these parameters does not invalidate the findings, it may have limited a more comprehensive understanding of the clinical contributors to thirst. Future studies incorporating these dialysis-specific variables alongside patient-reported outcomes are recommended to further elucidate the multifactorial nature of thirst in hemodialysis patients.

## 5. Conclusions

This study demonstrates that thirst in hemodialysis patients is not merely a physiological sensation but a multidimensional and complex experience encompassing physical, psychological, and behavioural dimensions. Quantitative findings revealed substantial thirst-related discomfort, as reflected by elevated Thirst Discomfort Scale and VAS scores, while qualitative findings highlighted symptoms such as dry mouth, excessive thirst, dizziness, weakness, fatigue, anxiety, and social withdrawal before and after dialysis.

These findings indicate that thirst cannot be effectively managed through fluid restriction alone. Instead, individual-centred, evidence-based, and holistic approaches are required to address both the physiological and experiential aspects of thirst. In this context, nurses play a pivotal role as the healthcare professionals closest to the patient, with responsibilities including early identification of thirst, planning patient-specific preventive strategies, providing education, and offering ongoing counselling.

From a clinical perspective, strengthening nursing interventions focused on patient education, counselling, and the teaching of effective coping strategies is essential. Practical measures such as evenly distributing fluid intake, low-sodium dietary counselling, and selected non-pharmacological approaches (e.g., ice cubes, sugar-free gum, oral rinsing) may be considered as supportive strategies in clinical practice. In addition, supporting fluid monitoring and patient education with technological solutions, such as mobile applications and digital reminders, may further support patient comfort, self-management, and engagement in care processes.

Overall, integrating patients’ lived experiences into nursing care and clinical decision-making may inform the development of evidence-based protocols for thirst management and support patient comfort and self-management in hemodialysis care. Future research should build on these findings through longitudinal designs to examine changes in thirst experiences over time, multicenter studies to enhance generalizability, and intervention studies evaluating the effectiveness of specific nursing-led coping strategies on thirst-related discomfort and patient outcomes.

## Figures and Tables

**Table 1:** Distribution of demographic characteristics of patients receiving hemodialysis treatment.

Features		*n*	%
**Body Mass Index (BMI)**	<18.5	8	11.1
	18.5–24.9	28	38.9
	>24.9	36	50.0
**Gender**	Female	36	50
	Male	36	50
**Marital Status**	Married	69	95.8
	Single	3	4.2
**Diabetes Mellitus**	Yes	35	48.6
	None	37	51.4
**Hypertension**	Yes	58	80.6
	None	14	19.4
**Mobility**	Independent	61	84.7
	Support	7	9.7
	Dependent	4	5.6
**Wound/Abrasion in the mouth**	Yes	4	5.6
	None	68	94.4
**Skin Dryness Status**	Yes	55	76.4
	None	17	23.6
**Itching Condition**	Yes	42	58.3
	None	30	41.7
		**Mean ± SD**	**Min–Max**
**Age (Year)**		59.15 ± 13.70	20–80
**Daily tea/coffee consumption (cups)**		3.22 ± 1.59	0–10
**Daily Liquid Intake (Glass)**		3.92 ± 2.17	1–10
**Hemodialysis Treatment Duration (Months)**		48.35 ± 47.0	1–180
**VAS Thirst Score (0–10)**		7.50 ± 1.82	2–10

**Table 2:** Distribution of the mean total scores of the Thirst Discomfort Scale and its sub-dimensions.

	Mean ± SD	Min–Max
**Intraoral Movements (IM)**	18.04 ± 3.80	10–25
**Psychological Movements (PM)**	14.88 ± 2.75	8–20
**Extraoral Movements (EOM)**	11.55 ± 2.87	6–15
**Thirst Discomfort Scale Total (TDS)**	44.48 ± 7.90	25–57

**Table 3:** Themes, sub-themes, and codes obtained from interviews with patients receiving hemodialysis treatment.

Theme	Sub Theme	Code	Participants	*n*
**Effects of Hemodialysis on Thirst and Problems Experienced**	**Psychological** **Impacts**	Fatigue	1, 5, 6, 7, 19, 20, 22	7
		Fear	3, 4, 14, 15, 18, 20	6
		Failure to socialise	3, 4, 6, 21	4
	**Pre-dialysis Problems**	Dry mouth/throat	3, 5, 6, 7, 8, 10, 11, 12, 13, 14, 18, 22	12
		Inability to drink water	3, 4, 7, 8, 9, 14, 16, 19, 20, 22	10
		Excessive thirst	1, 2, 3, 5, 9, 11, 13, 16, 17	9
		Bloating/Difficulty in movement	1, 8, 11, 12, 13, 16, 18	7
		Inability to excrete	1, 3, 11, 16	4
	**Problems After Dialysis**	Dry mouth	1, 3, 4, 5, 6, 7, 8, 11, 12, 13, 14, 16, 18, 19, 20, 21, 22	17
		Excessive thirst	3, 4, 5, 6, 7, 10, 11, 13, 15, 16	10
		Dizziness/weakness	7, 12, 13, 14, 18, 20, 21, 22	8
		Drop in blood pressure	5, 6, 10, 12, 13	5
**Time, Factors, and Coping Experiences with Thirst**	**Period of Thirst**	After dinner	1, 2, 4, 8, 9, 10, 12, 14, 15, 16, 18, 20, 22	13
		Night	2, 3, 4, 5, 6, 8, 10, 13, 14, 15, 16	11
		Evening	2, 4, 6, 9, 10, 12, 14, 21, 22	9
		Morning	3, 5, 6, 11, 17, 19, 20, 21	8
		Noon	1, 6, 7, 15, 18, 21, 22	7
		Summer months	10, 13, 14, 15, 21	5
	**Factors Increasing Thirst**	Movement	8, 9, 10, 12, 13, 14, 17, 18, 21	9
		Fatigue	4, 5, 10, 13, 18	5
		Salt intake	3, 4, 12, 14, 17	5
		Meat consumption	1, 7, 9, 12, 16	5
	**What to do to quench thirst**	Drinking cold water	2, 3, 4, 8, 9, 10, 11, 12, 15, 16, 19, 20, 21	13
		Eating fruit	1, 2, 3, 4, 10, 12, 18, 20, 21, 22	10
		Tea drinking	6, 12, 14, 15, 16, 18, 19, 20, 22	9
		Rinsing the mouth with water	3, 5, 10, 13, 15, 16	6
		Drinking buttermilk	7, 11, 12, 13, 20	5

## Data Availability

The data in this study cannot be used due to confidentiality or ethical constraints.

## Abbreviations

The following abbreviations are used in this manuscript:

CRF	Chronic Renal Failure
IDWGA	Interdialytic Weight Gain
VAS	Visual Analogue Scale
BMI	Body Mass Index
